# Synthesis and Study of Fe-Doped Bi_2_S_3_ Semimagnetic Nanocrystals Embedded in a Glass Matrix

**DOI:** 10.3390/molecules22071142

**Published:** 2017-07-11

**Authors:** Ricardo S. Silva, Hanna D. Mikhail, Eder V. Guimarães, Elis R. Gonçalves, Nilo F. Cano, Noelio O. Dantas

**Affiliations:** 1 Departamento de Física, Instituto de Ciências Exatas, Naturais e Educação (ICENE), Universidade Federal do Triângulo Mineiro, Uberaba 38025–180, Minas Gerais, Brazil; edervgol@gmail.com (E.V.G.); gelisregina@gmail.com (E.R.G.); 2 Departamento de Engenharia Mecânica, Instituto de Ciências Tecnológicas e Exatas (ICTE), Universidade Federal do Triângulo Mineiro, Uberaba 38064–200, Minas Gerais, Brazil; hanna.mikhail@uftm.edu.br; 3 Instituto do Mar, Universidade Federal de São Paulo, Santos 11070–100, São Paulo, Brazil; nilo.cano@unifesp.br; 4 Laboratório de Novos Materiais Isolantes e Semicondutores (LNMIS), Instituto de Física, Universidade Federal de Uberlândia, Uberlândia 38400–902, Minas Gerais, Brazil; noelio@ufu.br

**Keywords:** Fe-doped bismuth sulphide, nanocrystal synthesis, density functional theory

## Abstract

Iron-doped bismuth sulphide (Bi_2−*x*_Fe*_x_*S_3_) nanocrystals have been successfully synthesized in a glass matrix using the fusion method. Transmission electron microscopy images and energy dispersive spectroscopy data clearly show that nanocrystals are formed with an average diameter of 7–9 nm, depending on the thermic treatment time, and contain Fe in their chemical composition. Magnetic force microscopy measurements show magnetic phase contrast patterns, providing further evidence of Fe incorporation in the nanocrystal structure. The electron paramagnetic resonance spectra displayed Fe^3+^ typical characteristics, with spin of 5/2 in the 3d^5^ electronic state, thereby confirming the expected trivalent state of Fe ions in the Bi_2_S_3_ host structure. Results from the spin polarized density functional theory simulations, for the bulk Fe-doped Bi_2_S_3_ counterpart, corroborate the experimental fact that the volume of the unit cell decreases with Fe substitutionally doping at Bi1 and Bi2 sites. The Bader charge analysis indicated a pseudo valency charge of 1.322|*e*| on Fe_Bi__1_ and 1.306|*e*| on Fe_Bi__2_ ions, and a spin contribution for the magnetic moment of 5.0 *µ_B_* per unit cell containing one Fe atom. Electronic band structures showed that the (indirect) band gap changes from 1.17 eV for Bi_2_S_3_ bulk to 0.71 eV (0.74 eV) for Bi_2_S_3_:Fe_Bi1_ (Bi_2_S_3_:Fe_Bi2_). These results are compatible with the 3d^5^ high-spin state of Fe^3+^, and are in agreement with the experimental results, within the density functional theory accuracy.

## 1. Introduction

Semiconductor nanocrystals doped with transition metals constitute a class of new nanomaterials that have been intensively investigated recently, mainly due to their interesting and tunable physical properties, which are significantly different from that of the nonmagnetic host semiconductor nanocrystal [1,2,3,4]. These materials, in which the magnetic dopant concentration is typically a few percentage points, are known as diluted magnetic semiconductors (DMS). The main cause of the new physical properties owned by these DMS materials is attributed to the sp- exchange interaction between the host nonmagnetic semiconductor sp-band and the partially occupied transition metal d-state [5,6,7]. Synthesis of several kinds of transition metal doped semiconductor nanocrystals are reported in the literature, such as Mn-doped Bi_2_S_3_ [8] and PbSe [9], Cu-doped SrF_2_ [10], Co-doped PbSe [11], Cr-doped In_2_O_3_ [12], and Fe-doped ZnO [13], PbTiO_3_ [14], and TiO_2_ [15]. These DMS nanocrystals can be applied in light-emitting diodes (LEDs) [16], solar cells [17], optical ratio-metric temperature sensors [18], and spintronic devices [19].

One material that has potential technological applications to be explored, and which present interesting electronic properties, is the bismuth sulphide (Bi_2_S_3_) semiconductor [20,21]. For Bi_2_S_3_ bulk, it has been reported a direct band gap of 1.3–1.7 eV [22,23,24], from both experimental and theoretical methods with the framework of the GW DFT (Green function and screened coulomb interaction), whereas standard DFT reports indirect band gap of 1.19 eV [25]. Bi_2_S_3_ crystallizes in the orthorhombic structure (*Pnma* space group), with a unit cell consisting of four Bi_2_S_3_ units, leading to a 20 atoms unit cell [26]. Each Bi_2_S_3_ unit has two nonequivalent Bi sites, denoted as Bi1 and Bi2, which differs in their coordination, as well as three nonequivalent S sites, denoted as S1–S3.

The electronic properties of semiconductor nanocrystals can be tunable by means of quantum confinement effects, as well as by doping with magnetic ions [27,28]. Therefore, doping Bi_2_S_3_ nanocrystals with Fe ions could lead to physical properties tunable by size and dopant concentration control. As Fe ions enter into the Bi_2_S_3_ structure, most probably as Fe^3+^ substituting Bi^3+^ ions, with 3d^5^ valence configuration and spin of 5/2 [29], it became possible to add magnetic properties to the nonmagnetic Bi_2_S_3_ nanocrystals. The exchange interaction between the sp-band from the Bi_2_S_3_ semiconductor, and the d-state from the Fe^3+^ ion become important for their electronic and, consequently, optical properties.

In this work, we report the synthesis and study of iron-doped bismuth sulphide (Bi_2−*x*_Fe*_x_*S_3_) nanocrystals embedded in a host glass matrix (referred to as SNAB) by the fusion method, with Fe-concentration of *x* = 0.00, *x* = 0.05, and *x* = 0.10. The SNAB glass matrix was chosen because it presents good chemical stability, optical transparency in the visible and near-infrared spectra regions, and is nontoxic, which makes it an excellent template for the growth of different kinds of nanocrystals [8,30,31,32]. The structural and magnetic properties of Bi_2−*x*_Fe*_x_*S_3_ nanocrystals were investigated experimentally by the techniques of transmission electron microscopy (TEM), energy dispersive spectroscopy (EDS), atomic and magnetic force microscopy (AFM/MFM), and electron paramagnetic resonance (EPR). In order to gain insight into the Fe-doping effect on the structural parameters of Bi_2_S_3_ nanocrystals, a theoretical study was performed on Fe-doped bulk Bi_2_S_3_, based on density functional theory (DFT), using pseudopotentials and numerical atomic basis set.

## 2. Results and Discussion

**Figure 1:**
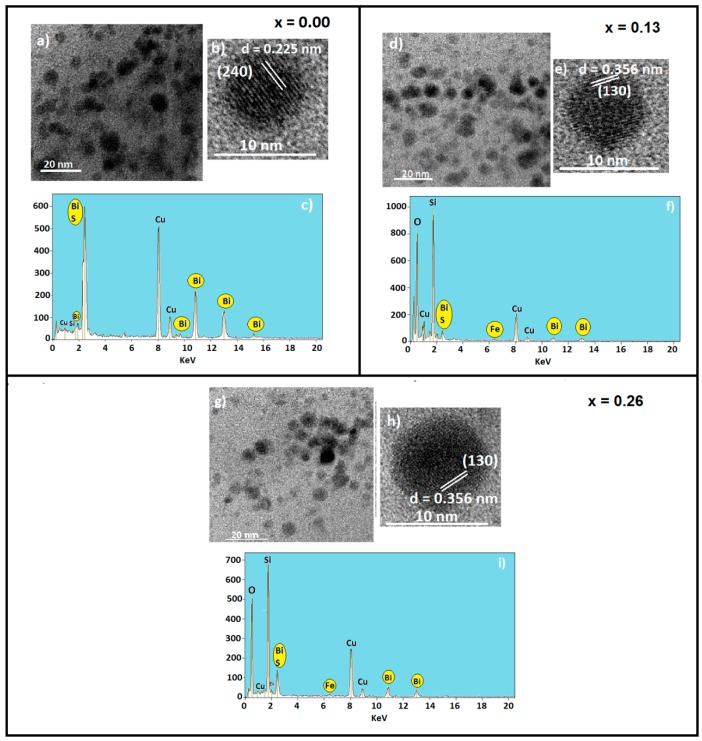
TEM images of Bi_2−*x*_Fe*_x_*S_3_ nanocrystals grown in the SNAB glass matrix with mean size of 7.0 nm, for *x* values: (**a**) 0.00; (**d**) 0.13; and (**g**) 0.26 and from 9.0 nm to the values of *x*: (**b**) 0.00; (**e**) 0.13; and (**h**) 0.26. EDS measurements for samples treated at 500 °C for 10 h are given for *x* values: (**c**) 0.00; (**f**) 0.13; and (**i**) 0.26. The white circle in the TEM images indicates the region in which the EDS measurement was performed in the samples.

TEM images of Bi_2−*x*_Fe*_x_*S_3_ nanocrystals growth in the SNAB glass matrix, and those thermally treated at 500 °C for 10 h, are displayed in Figure 1 for (a) *x* = 0.00, (d) *x* = 0.13, and (g) *x* = 0.26, as well as a correspondent nanocrystal magnified image for the samples thermally treated at 500 °C for 24 h (Figure 1b,e,h) and the EDS data (Figure 1c,f,i). An average diameter of 7 nm has been obtained. From the magnified TEM images (to samples treated for 24 h), the mean diameter of the single nanocrystal is 9 nm, the interplanar distance of *d*_240_ = 0.225 nm (*x* = 0.00) and *d*_130_ = 0.356 nm (*x* = 0.13 and 0.26) was estimated and attributed to the bulk Bi_2_S_3_ (240) and (130) crystalline planes, respectively. These estimations were done with the software ImageJ [33]. EDS measurements of the samples treated for 10 h, confirm the chemical composition of the Fe-doped Bi_2_S_3_ nanocrystals. The presence of Fe can be seen at about 6.4 keV in the EDS spectrum.

**Figure 2:**
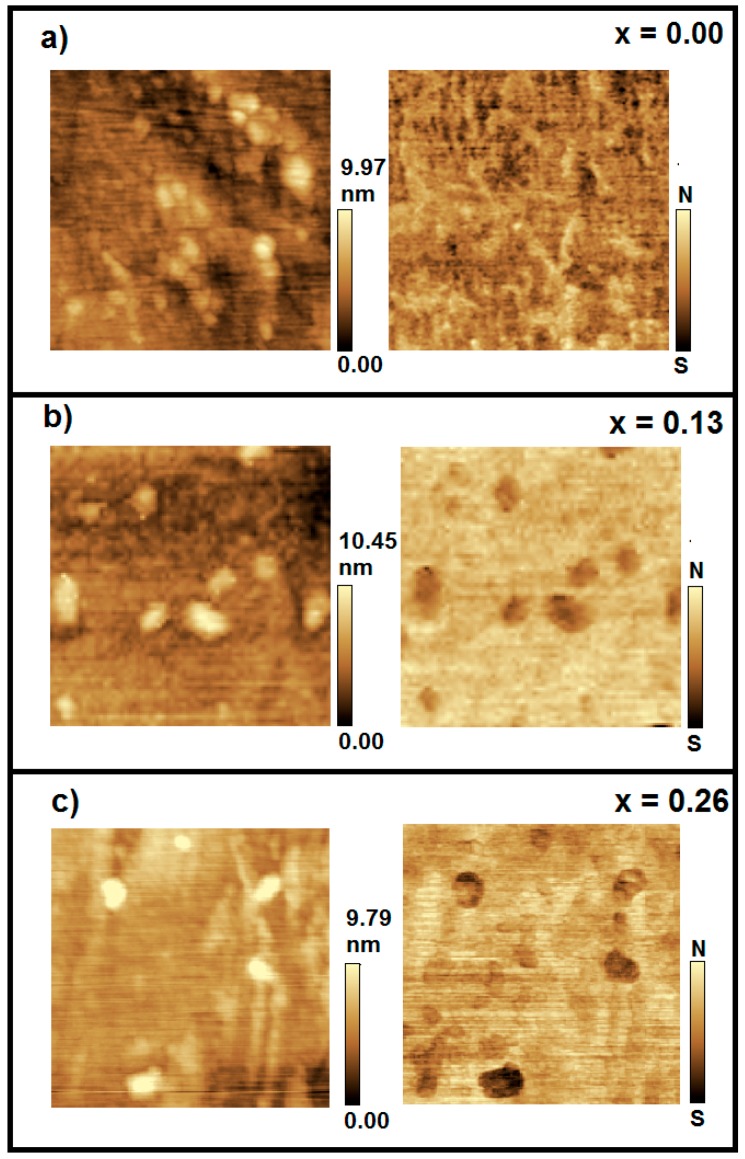
AFM/MFM images of 700 × 700 nm of Bi_2−*x*_Fe*_x_*S_3_ nanocrystals grown in the SNAB glass matrix, heat treated at 500 °C for 24 h, at concentrations: (**a**) *x* = 0.00; (**b**) *x* = 0.13; and (**c**) *x* = 0.26. Sample topographic (**left** panel) and magnetic phase (**right** panel) identifies the orientation of the total magnetic moment of the DMS NCs.

Figure 2 shows the AFM/MFM images (700 × 700 nm) used for magnetic investigations of Bi_2−*x*_Fe*_x_*S_3_ nanocrystals, with an average size of 9.0 nm (obtained by TEM images), being: (a) *x* = 0.00, (b) *x* = 0.13, and (c) *x* = 0.26. Topographic images are shown in the left panels, and corresponding magnetic phase images are shown in the right panels. The bright/dark contrast in the MFM magnetic phase images is attributed to the magnetic response of Bi_2−*x*_Fe*_x_*S_3_ nanocrystals when induced by a magnetized tip. The bright/dark contrast is due to the repulsion/attraction of the magnetized tip to the nanocrystals, represented in the vertical bar, as with the north (N) (south (S)) poles [34]. The same contrast does not appear for the sample containing only non-doped Bi_2_S_3_ nanocrystals (*x* = 0.00). These AFM/MFM images support TEM/EDS data and EPR measurements, providing evidence of the incorporation of Fe^3+^ magnetic ions into the crystalline structure of Bi_2−*x*_Fe*_x_*S_3_ nanocrystals.

EPR measurements were carried out at 300 K and at X-band frequencies on Bi_2−*x*_Fe*_x_*S_3_ nanocrystals grown in the SNAB matrix. Fe^3+^ ions (3d^5^, *S* = 5/2) have typical absorptions at *g*-factor equal to 4.30 and 2.00. In general, the spin Hamiltonian for Fe^3+^ ions is represented by [29]

$$H=g\mu_{B}H.S+D\left\{S_{z}^{2}+\left(\frac{S\left(S+1\right)}{3}\right)\right\}+E\left(S_{x}^{2}-S_{y}^{2}\right)$$

**Figure 3:**
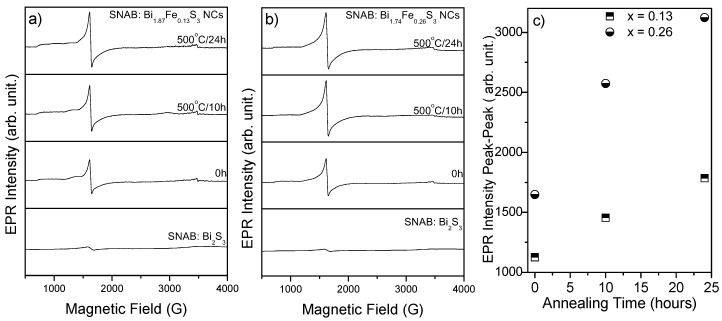
Bi_2−*x*_Fe*_x_*S_3_ nanocrystal EPR spectra grown in the SNAB glass matrix for: (**a**) *x* = 0.13 and (**b**) *x* = 0.26. SNAB: Bi_2_S_3_ is correspondent to the case where *x* = 0.00. In (**c**), the intensity variation of the EPR signal is shown for the samples without treatment and with treatment times of 10 and 24 h at 500 °C.

Here, *g* is gyromagnetic factor, $\mu_{B}$ is the Bohr magneton, *H* is the applied magnetic field, *S* the effective spin of the Fe^3+^ ion, S*_i_*(*i* = *x*, *y*, *z*) are the spin angular momentum operators in the coordinate system. The terms *D* and *E* are the second order terms of the crystalline field, with axial and rhombic symmetry, respectively, and represent the interaction with the zero magnetic field. The Hamiltonian spin of the Fe^3+^ ion is strongly dependent on the *D* value, providing a value of the *g*-factor close to 2.0023 when *D* smaller than $\mu_{B}H$, independent of E. However, when the term *D* is greater than $g\mu_{B}H$, the g-factor has values around *g* = 4.3 (*E* ≠ 0) and *g* = 6.0 (*E* = 0). In literature, the absorptions at *g* = 4.3 and *g* = 6.0 are attributed to the location of the Fe^3+^ ions in tetrahedral or octahedral sites [35,36]. In Figure 3, the EPR spectra are presented for the untreated samples and for samples treated at 500°C for 10 and 24 h. These spectra are for sample with (a) *x* = 0.13 and (b) *x* = 0.26. The annealing treatment increases the size of the Bi_2−*x*_Fe*_x_*S_3_ nanocrystals and, consequently, increases the intensity which is attributed to a greater incorporation of the Fe^3+^ ions, as substitution for the ions of Bi^3+^, in the crystalline structure of the Bi_2_S_3_ semiconductor. In the particular case of *x* = 0.00, the EPR signal of Fe^3+^ ions are not observed. This increase in the peak-to-peak intensity of the Fe^3+^ ion EPR signal is attributed to the influence of the crystalline field of the Bi_2_S_3_ nanocrystals and is analyzed in Figure 3c for the concentrations of *x* = 0.05 and 0.10. The EPR spectra amplitude was normalized by the mass of the samples. The EPR data is in agreement with the results obtained by TEM/EDS and AFM/MFM.

**Figure 4:**
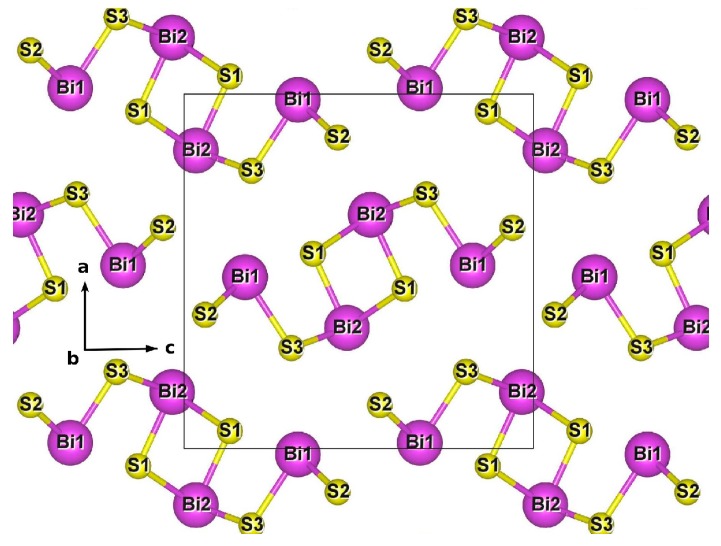
Crystal structure of bulk Bi_2_S_3_, at zero pressure, showing the unit cell.

The orthorhombic unit cell used in the DFT simulation of bulk Bi_2_S_3_ is illustrated in Figure 4. It consists of 8 Bi and 12 S atoms, adding up to 20 atoms per unit cell. The optimized lattice parameters and unit cell volume of bulk Bi_2_S_3_ crystal structure at zero pressure are collected in Table 1, and are compared with the correspondent experimental values from Lundegaard et al. [26]. The calculated lattice parameters and unit cell volume for bulk Bi_2_S_3_ crystal structure are in excellent agreement with the experimental ones, within the DFT-GGA accuracy.

Data for crystal structures of Fe-doped Bi_2_S_3_ are also collected in Table 1 for Fe occupying Bi1 or Bi2 sites. The results show that when bulk Bi_2_S_3_ is doped with Fe, the unit cell decreases in volume by a factor of 3.6% for Fe_Bi1_ and of 1.9% for Fe_Bi2_. Total energy calculations show that Fe_Bi1_ structure is energetically favorable relative to Fe_Bi2_ by only 289 meV, which means that both Bi sites are almost just as likely to be occupied by Fe atoms. Results from Bader charge analysis [42,43] are displayed in Table 2, showing a pseudo valency charge of 1.322|*e*| on Fe_Bi__1_ and 1.306|*e*| on Fe_Bi2_ ions, which compares well with the pseudo valency charge of 1.454|*e*| and 1.600|*e*| on Bi1 and Bi2 sites at Bi_2_S_3_ undoped structure, with small charge transfer among atoms. For Fe-doped Bi_2_S_3_ bulk, Bader charges and pseudo valency charges on different Bi and S sites change very little and are not shown in Table 2. For both Fe-doped structures, a spin contribution for the magnetic moment of 5.0 *μ_B_* per unit cell containing one Fe atom was obtained.

**Figure 5:**
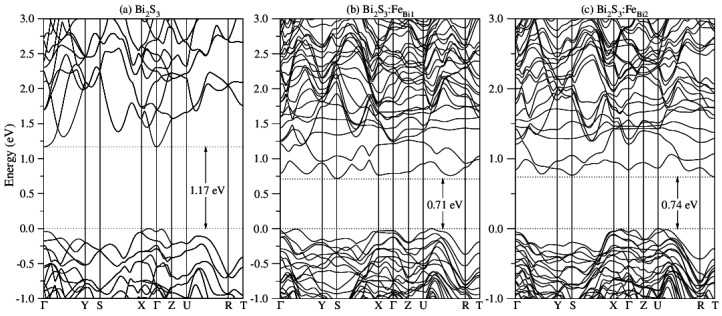
First-principle band structures for (**a**) bulk Bi_2_S_3_, (**b**) Fe-doped Bi_2_S_3_ at Bi1 site, and (**c**) Fe-doped Bi_2_S_3_ at Bi2 site. Horizontal dashed lines indicate valence bands maximum (set to zero) and conduction bands minimum. The selected *k* points are Γ = (0,0,0), Y = (0,½,0), S = (½,½,0), X = (½,0,0), Z = (0,0, ½), U = (½,0,½), R = (½, ½, ½), and T = (0,½,½).

The energy band structure of Bi_2_S_3_ bulk and Fe-doped Bi_2_S_3_, along the high symmetry directions in the Brillouin zone, are shown in Figure 5. For Bi_2_S_3_, the band structure (Figure 5a) indicate an indirect band gap of 1.17 eV, with valence band maximum (VBM) within the X–Γ line and conduction band minimum (CBM) at the Γ point, which is in good agreement with other theoretical calculations [25]. Iron doping results in a reduction of the band gap to 0.71 eV for Fe atoms occupying Bi1 sites (Figure 5b) and to 0.74 eV for Fe atoms occupying Bi2 sites (Figure 5c), due to energy levels introduced by Fe orbitals. 

## 3. Materials and Methods

Bi_2−*x*_Fe*_x_*S_3_ nanocrystal samples were synthesized by the fusion method in a borosilicate glass matrix, referred to as SNAB, with the following nominal composition: 45SiO_2_·30Na_2_CO_3_·5Al_2_O_3_·20B_2_O_3_ (mol %), and adding 2% of Bi_2_O_3_ and S (wt), and nominal *x* content of Fe (*x* = 0.00, 0.13, and 0.26) as a function of bismuth concentration. The powder mixture of the glass and the nanocrystal precursors were combined and melted in an alumina crucible, at 1200 °C for 30 min, and then rapidly cooled at room temperature. The resulting samples were then thermally treated in ambient air at 500 °C for 10 and 24 h to provide the energy and time needed for the diffusion of the Bi^3+^, S^2−^, and Fe^3+^ ions throughout the host matrix. This annealing process produces Fe-doped Bi_2_S_3_ nanocrystals with small size distribution. TEM micrographs and EDS were taken using a JEM-2100 (JEOL, 200 kV) to investigate the formation, size, shape, and growth of the Bi_2−*x*_Fe*_x_*S_3_ nanocrystals. Since TEM images of dielectric materials (glass template) are difficult to obtain, the samples were turned into a finer powder, and placed on a plate made of copper in order of take the TEM measurements. AFM/MFM images were recorded in a Shimadzu (SPM-9600) scanning probe microscope, with nominal resolution in the vertical direction for topographic mode of 0.01 nm and horizontally of 0.2 nm. Iron magnetic impurity electronic states, in the structure of the doped nanocrystals, were studied via EPR, using a ST ER4102 spectrometer (Bruker EMX spectrometer) with a rectangular cavity, microwave frequency of 9.75 GHz (X-band), microwave power of 20 mW, and 100 kHz field modulation. All measurements were taken at room temperature.

In order to obtain the structural effect, the Bader charge analysis, and the spin contribution to the magnetic moment of Fe-doped Bi_2_S_3_ nanostructure, first principle simulations based on density functional theory [37,38] were carried out. All the simulations were performed using norm-conserving pseudopotentials [39] and the PBE generalized gradient approximation [40] for the electronic exchange-correlation functional, as implemented in the Siesta [41] code. Relativistic pseudopotentials, chosen to enable spin polarized simulations (without taking into account the spin-orbit interaction), were generated with the following valence configurations: 6s^2^ 6p^3^ 6d^0^ 5f^0^ for Bi, 3s^2^ 3p^4^ 3d^0^ 4f^0^ for S, and 3d^6^ 4s^2^ 4p^0^ 4f^0^ for Fe.

One conventional unit cell of Bi_2_S_3_, containing 20 atoms (four stoichiometric Bi_2_S_3_ formulas) was used as a supercell. The experimental data for bulk Bi_2_S_3_ [26] was taken as the starting geometry. Then, full relaxation of lattice parameters and atomic coordinates were performed. For Fe-doped Bi_2_S_3_ simulations, starting from the full relaxed Bi_2_S_3_ bulk structure, one Bi atom (at Bi1 or Bi2 site) was substituted by one Fe atom per unit cell and fully relaxed again, getting an Fe atomic doping concentration of 12.5%. Indeed, this is a higher concentration, but it is near to the experimental concentration of the sample synthesized with 13.0% of Fe (nominal) concentration.

The following parameters were used for all the calculations: a DZP basis set with an energy shift of 100 meV, a mesh cutoff of 250 Ry (~3400 eV), a maximum difference tolerance in the density matrix of 1 × 10^−5^, a tolerance in the total free energy of 1 × 10^−4^ eV, 0.01 eV/Å for the force relaxation criterion, 0.05 GPa as the maximum difference in the stress tensor components, and 36 k-points in the unit cell for k-sampling in the Brillouin zone. Only for Bader charge analysis, a mesh cutoff of 300 Ry was set.

## 4. Conclusions

Diluted magnetic semiconductor nanocrystals of Bi_2−*x*_Fe*_x_*S_3_ have been successfully synthesized in a glass matrix by the fusion method. TEM measurements confirm the formation of nanocrystals with average diameter of 7 nm and 9 nm for samples thermally treated at 500 °C for 10 and 24 h, respectively. EDS measurement suggested that the Fe ions were incorporated in the nanocrystal structure, which is confirmed from the observed magnetic phase contrast in the AFM/MFM measurements, which is attributed to the magnetic response of Fe^3+^ ions in the nanocrystal structure. EPR spectra confirm the Fe^3+^ valence state with a spin of 5/2, and the crystalline field influence on the Bi_2_S_3_ nanocrystals. DFT simulations showed that when bulk Bi_2_S_3_ is doped with Fe, the unit cell decreases in volume by a factor of 3.6% for Fe_Bi__1_ and of 1.9% for Fe_Bi2_, and that the Fe_Bi1_ structure is energetically favorable to Fe_Bi2_ by 289 meV. Bader charge analysis indicated a pseudo valency charge of 1.322|*e*| on Fe_Bi1_ and 1.306|*e*| on Fe_Bi2_ ions, and a spin contribution for the magnetic moment of 5.0 *μ_B_* per unit cell containing one Fe atom, for both Fe_Bi1_ and Fe_Bi2_ doped structures. Calculated electronic band structures showed that the indirect band gap changes from 1.17 eV for Bi_2_S_3_ bulk to 0.71 eV (0.74 eV) for Bi_2_S_3_:Fe_Bi1_ (Bi_2_S_3_:Fe_Bi2_).

## Figures and Tables

**Table 1:** Calculated zero pressure lattice parameters and unit cell volumes for bulk Bi_2_S_3_, Fe-doped Bi_2_S_3_ at Bi1 and at Bi2 sites. Experimental data for bulk Bi_2_S_3_ at zero pressure was taken from Ref. [26]. For all the structures, *α* = *β* = *γ* = 90°.

Structural Parameters	Bi_2_S_3_ Exp.[26]	Bi_2_S_3_	Bi_2_S_3_:Fe_Bi1_	Bi_2_S_3_:Fe_Bi2_
a (Å)	11.282	11.249 dev. −0.29%	11.419	11.229
b (Å)	3.9728	4.0296 dev. 1.43%	3.9540	3.9318
c (Å)	11.131	11.004 dev. −1.14%	10.652	11.077
V*_uc_* (Å^3^)	498.4	498.8 dev. 0.08%	480.9	489.1

**Table 2:** Bader charge calculated from pseudo valency electron density for Bi_2_S_3_ bulk, Fe-doped Bi_2_S_3_ bulk at Bi1 and at Bi2 sites. Pseudo valency charge is the difference between the free atom valency charge and the Bader charge. All charges in units of |*e*|, where *e* is the elementary charge.

Structure	Atom	Bader Charge	Pseudo Valency Charge
Bi_2_S_3_	Bi1	3.546	1.454
	Bi2	3.400	1.600
	S1	7.108	−1.108
	S2	6.986	−0.986
	S3	6.960	−0.960
Bi_2_S_3_:Fe_Bi1_	Fe1	6.678	1.322
Bi_2_S_3_:Fe_Bi2_	Fe2	6.694	1.306

## Supplementary Materials

Supplementary File 1
