# Supplementary figures and images for: Synthesis and Study of Fe-Doped Bi2S3 Semimagnetic Nanocrystals Embedded in a Glass Matrix
Source: Molecules. 2017 Jul 11;22(7):1142. doi: 10.3390/molecules22071142 (PMC6152244; doi:10.3390/molecules22071142)

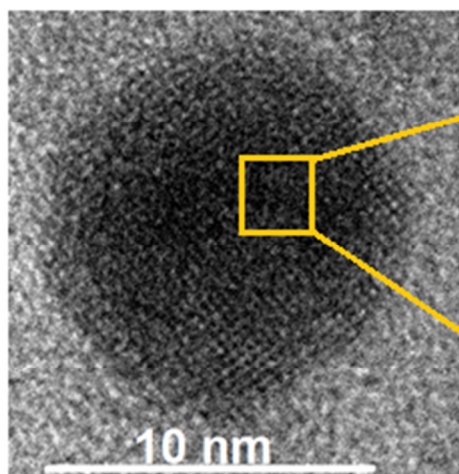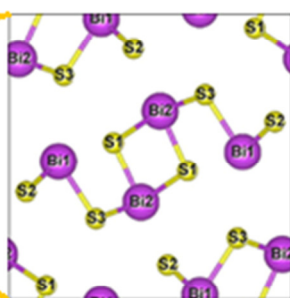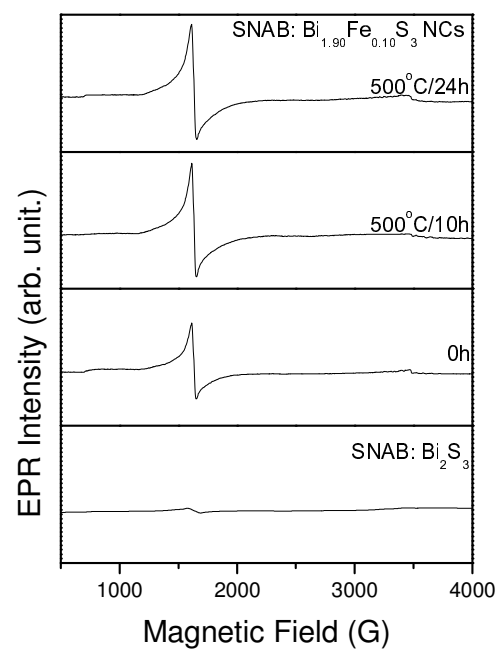

Supplement: Supplementary File 1 [file molecules-22-01142-s001.pdf]
